# What is ‘moral distress’? A narrative synthesis of the literature

**DOI:** 10.1177/0969733017724354

**Published:** 2017-10-08

**Authors:** Georgina Morley, Jonathan Ives, Caroline Bradbury-Jones, Fiona Irvine

**Affiliations:** University of Bristol, UK; University of Birmingham, UK

**Keywords:** Bioethics, empirical approaches, literature review, moral distress, narrative synthesis, nursing ethics, nursing

## Abstract

**Aims:**

The aim of this narrative synthesis was to explore the necessary and sufficient conditions required to define moral distress.

**Background:**

Moral distress is said to occur when one has made a moral judgement but is unable to act upon it. However, problems with this narrow conception have led to multiple redefinitions in the empirical and conceptual literature. As a consequence, much of the research exploring moral distress has lacked conceptual clarity, complicating attempts to study the phenomenon.

**Design:**

Systematic literature review and narrative synthesis (November 2015–March 2016).

**Data sources:**

Ovid MEDLINE^®^ In-Process & Other Non-Indexed Citations 1946–Present, PsycINFO^®^ 1967–Present, CINAHL^®^ Plus 1937–Present, EMBASE 1974–24 February 2016, British Nursing Index 1994–Present, Social Care Online, Social Policy and Practice Database (1890–Present), ERIC (EBSCO) 1966–Present and Education Abstracts.

**Review methods:**

Literature relating to moral distress was systematically retrieved and subjected to relevance assessment. Narrative synthesis was the overarching framework that guided quality assessment, data analysis and synthesis.

**Results:**

In all, 152 papers underwent initial data extraction and 34 were chosen for inclusion in the narrative synthesis based on both quality and relevance. Analysis revealed different proposed conditions for the occurrence of moral distress: moral judgement, psychological and physical effects, moral dilemmas, moral uncertainty, external and internal constraints and threats to moral integrity.

**Conclusion:**

We suggest the combination of (1) the experience of a moral event, (2) the experience of ‘psychological distress’ and (3) a direct causal relation between (1) and (2) together are necessary and sufficient conditions for moral distress.

## Introduction

The concept of moral distress (MD) was introduced to nursing by Jameton^1^ who defined MD as arising, ‘when one knows the right thing to do, but institutional constraints make it nearly impossible to pursue the right course of action’. MD has subsequently gained increasing attention in nursing research, the majority of which conducted in North America but now emerging in South America, Europe, the Middle East and Asia. Studies have highlighted the deleterious effects of MD, with correlations between higher levels of MD, negative perceptions of ethical climate^2^ and increased levels of compassion fatigue among nurses.^3,4^ Consensus is that MD can negatively impact patient care, causing nurses to avoid certain clinical situations and ultimately leave the profession.^5,6^ MD is therefore a significant problem within nursing, requiring investigation, understanding, clarification and responses. The growing body of MD research, however, is arguably failing to bring the required clarification but rather has complicated attempts to study it.^7^ The increasing number of cited causes and effects of MD means the term has expanded to the point that according to Hanna^8^ and McCarthy and Deady,^9^ it is becoming an ‘umbrella term’ that lacks conceptual clarity referring unhelpfully to a wide range of phenomena and causes. Without, however, a coherent and consistent conceptual understanding, empirical studies of MD’s prevalence, effects, and possible responses are likely to be confused and contradictory.

A useful starting point is a systematic exploration of existing literature to critically examine definitions and understandings currently available, interrogating their similarities, differences, conceptual strengths and weaknesses. This article presents a narrative synthesis that explored proposed necessary and sufficient conditions for MD, and in doing so, this article also identifies areas of conceptual tension and agreement.

The language of necessary and sufficient conditions is commonly used in philosophy to define and explain connections between concepts and causality; offering a helpful way to conceptually examine MD. Mackie^10^ used the example of a house fire to explain the relationship between necessary and sufficient conditions, pointing out that there is no single necessary and sufficient condition for a house fire, but there are some necessary conditions for a fire to occur (such as heat, oxygen, combustible material) and there are various groups of conditions that are sufficient together but not necessary (a match, for example, can cause a fire, but so too can a lighter). Importantly, there cannot be a house fire unless these necessary and sufficient conditions (sources of ignition, combustible material, oxygen) are met.

To use a different illustration, there are certain conditions, such as frailty, immobility or poor nutrition, associated with the increased likelihood of developing a pressure ulcer. None of these, however, are necessary or sufficient conditions for a pressure ulcer; they are only factors that increase the likelihood of an ulcer forming when the necessary and sufficient conditions are met. There is one condition that is both necessary and sufficient for a pressure ulcer, and that is the presence of continued pressure on the skin. It is necessary because a pressure ulcer cannot occur without it, and it is sufficient because that is the only thing needed to form a pressure ulcer. One of the main challenges of defining MD is that, as we shall see, MD tends to be conceptualized in terms of the conditions in which it arises, for example, ‘MD occurs when conditions X and Y are met’. This is the starting point of our inquiry, with Jameton’s^1^ definition being framed in terms of the conditions in which MD arises. For Jameton, MD occurred when (1) a moral judgement has been made and (2) there are institutional constraints that prevent that moral judgement from being acted on. On this account, the presence of ‘constrained moral judgement’ is both a necessary and sufficient condition of MD. It is necessary because MD cannot occur without it, and it is sufficient because nothing else is needed for MD to occur. Literature on MD since then has either

1. Accepted this account of MD, as based on a single necessary and sufficient condition and defined in terms of that condition.
2. Challenged the necessity and/or sufficiency of that single condition.
3. Suggested adding other necessary or sufficient conditions.
4. Added to the necessary and sufficient conditions a range of specific causes of those conditions.

Definitions of MD are, therefore, a relatively confused and complex bundle of necessary and sufficient conditions, causes and effects, which the review reported in this article aims to unpick.

## Aims

The narrative synthesis aimed to identify key proposed definitions of MD and explore what components of those definitions, if any, ought to be considered necessary and/or sufficient conditions for MD.

## Methods

There are numerous kinds of systematic review, and our approach allows us to capture the rich and sometimes controversial conceptual development of MD in a way that is not overly reductive.^11^ Strech et al.^12^ suggest a seven-step systematic review process that is sensitive to the challenges of examining bioethical concepts such as MD. Combined with ‘narrative synthesis’, it provided the flexibility to systematically search the literature and synthesize key findings from a range of studies with different aims and using different methodologies. We used guidelines from Popay et al.^13^ to direct critical appraisal, data extraction and explore relationships between studies.

1. Defining the review question

The review question asked, simply, ‘how is MD defined or conceptualized?’ The search was not limited to one specific discipline because MD is not limited to healthcare or nursing. Table 1 provides details of the search strategy used for each discipline.

**Table 1.** Search strategy.

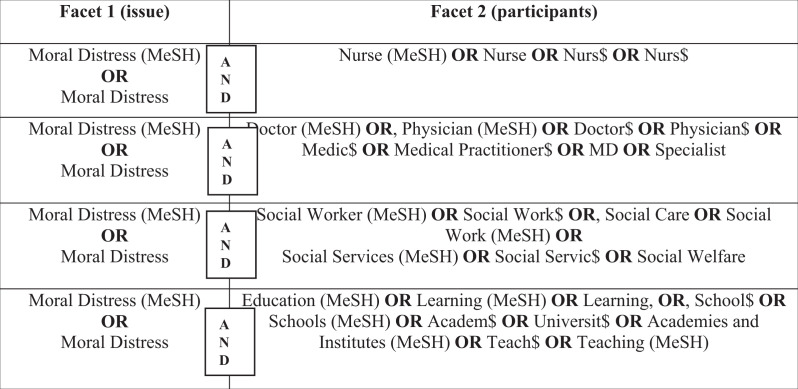

2. Selection of the relevant databases

Databases were searched according to the specific area under exploration. The following databases were searched: Ovid MEDLINE^®^ In-Process & Other Non-Indexed Citations 1946–Present; PsycINFO^®^ 1967–Present; CINAHL^®^ Plus 1937–Present; EMBASE 1974–24 February 2016; British Nursing Index 1994–Present; Social Care Online and Social Policy and Practice Database (1890–Present); ERIC (EBSCO) 1966–Present; Education Abstracts, cross referenced with EthxWeb (1974–2009) and EUROETHICS.

3. Ancillary search strategies

In addition to electronic searching on relevant databases, hand-searching reference of included studies was conducted to identify any overlooked papers.

4. Development of search algorithm

**Figure 1.**
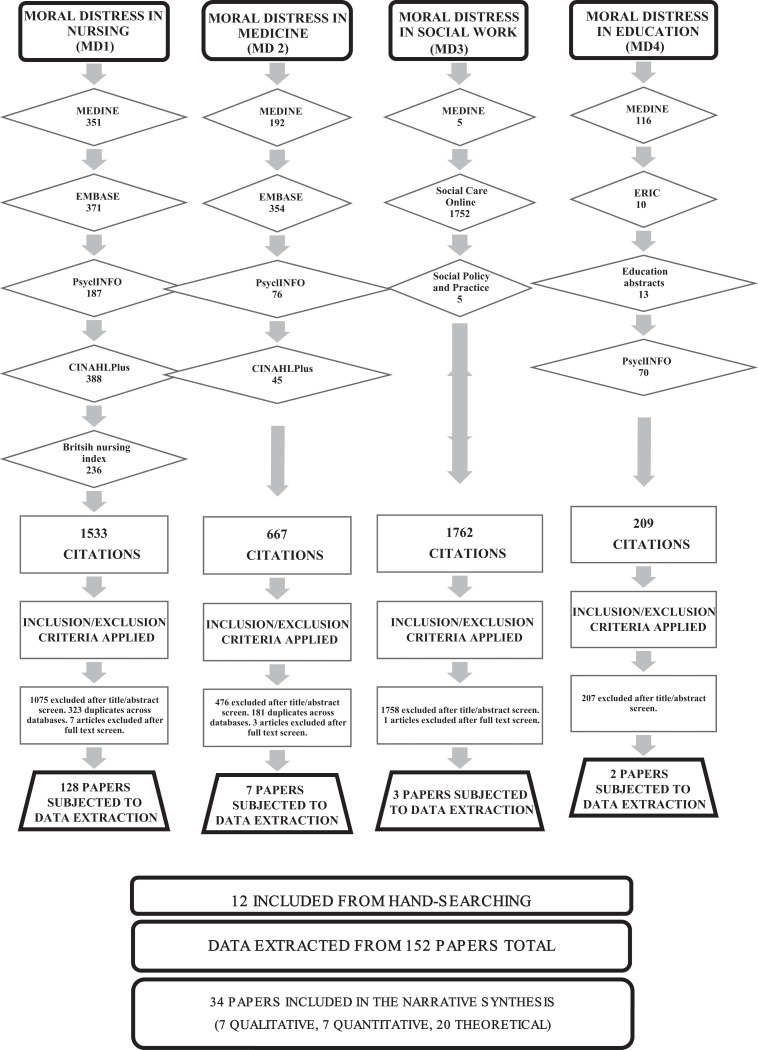
PRISMA table of search results.

Search terms and selection of relevant databases were guided by the discipline in which MD was being explored (nursing, medicine, social work, education). This process is detailed in the PRISMA diagram in Figure 1.

5. Relevance assessment of the retrieved references

**Figure 2.**
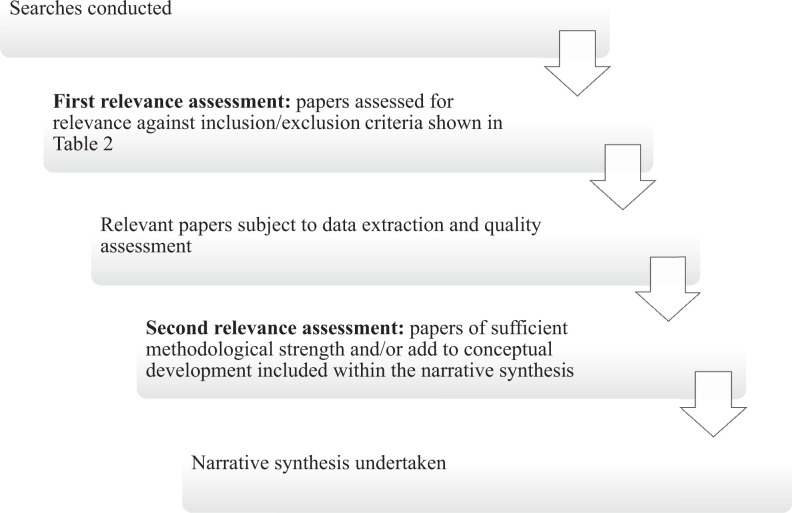
Flow diagram detailing the inclusion and exclusion process.

Relevance assessment was carried out at two stages (Figure 2). First, returned abstracts were reviewed for eligibility against the inclusion/exclusion criteria (Table 2). In total, 152 papers were selected for reading in full, data extraction and quality appraisal based on title and abstract review. Second, during quality appraisal, papers of insufficient quality, or those that did not meet inclusion criteria, were excluded.

**Table 2.** Inclusion and exclusion criteria for initial review.

Inclusion criteria	Exclusion criteria
Explores moral distress empirically. Explores moral distress conceptually or theoretically. Able to access an English language version.	Does not explore moral distress empirically. Does not explore moral distress conceptually. Moral distress is only mentioned in the discussion section. Editorials, letters or commentaries discussing moral distress. Intervention studies. Unable to access an English language version. Unpublished doctoral theses or dissertations.

6. Data extraction and quality appraisal

Quality assessment was undertaken on all papers that passed the first relevance assessment. Whittemore and Knafl^14^ note that quality appraisal in integrative reviews necessarily varies depending on the diversity of the included data, and as this review incorporated quantitative, qualitative and theoretical papers, this process was complex. Quality assessment was conducted using a critical appraisal and data extraction guide (adapted from Popay et al.^13^) that was adapted for use with both empirical and theoretical papers to inform thinking about the robustness of each paper. For example, quantitative papers were assessed for validity and rigour; qualitative papers were assessed for credibility and trustworthiness and theoretical or argument-based literature was assessed upon the strength, plausibility and transparency of arguments.^15^

Methodological quality influenced the second relevance assessment so that empirical papers deemed to be of poor quality were excluded (unless they also undertook theoretical analysis that contributed meaningfully to conceptual development). Conversely, papers judged to be methodologically strong but not providing conceptual insight were excluded (because they failed to meet inclusion criteria on that basis). After quality appraisal and relevance assessment (*n* = 34), papers were retained.

7. Data analysis and data presentation

Following Popay et al.,^13^ our synthesis involved four steps:

1. *Developing a ‘theory of change’*: The theory underpinning this review is that there needs to be a clear understanding of what MD is in order to avoid confusion and build a rigorous empirical base. In order to develop this theory, we began by exploring the commonly proposed/used definitions of MD (see Table 3).
2. *Developing a preliminary synthesis of findings from the included studies*: Textual descriptions of studies and tabulation was completed to gather preliminary information on all 152 studies but only those included in the narrative synthesis will be presented in this article (Table 4, Supplementary Material). Papers of sufficient methodological rigour and that provide conceptual insight (*n =* 34) are discussed and synthesized in the narrative below.
3. *Exploring relationships within and between studies*: Many of the studies explored causation or prevalence and because the aim was to gain a greater understanding of the phenomenon itself, charting of this specific data was deemed unnecessary. Conceptual relationships between studies were explored and tabulated.
4. *Assessing the robustness of the synthesis*: Assessing papers for conceptual development from diverse methodologies requires greater interpretation regarding relevance and consequently this step can be most susceptible to bias.^12^ In order to enhance trustworthiness, we have reported this process in a transparent manner.

**Table 3.** Common definitions of moral distress (chronological order).

No.	Reference	Definition	Necessary and/or sufficient conditions
1.	Jameton^1^	‘*Moral distress* arises when one knows the right thing to do, but institutional constraints make it nearly impossible to pursue the right course of action’.	Having made a moral judgement Institutional constraint Desired outcome may or may not be achieved
2.	Wilkinson^5^	‘Moral distress is defined by the author as the psychological disequilibrium & negative feeling state experienced when a person makes a moral decision but does not follow through by performing the moral behavior indicated by that decision’	Psychological effects Having made a moral decision Constraint on action Desired outcome not achieved
3.	Jameton^16^	‘…a nurse experiences moral distress when the nurse makes a moral judgment about a case in which he or she is involved and the institution or co-workers make it difficult or impossible for the nurse to act on that judgment’	Having made a moral judgement Institutional or coworker constraint Desired outcome may or may not be achieved
4.	Corley^17^	‘Jameton defined moral distress as painful feelings and/or psychological disequilibrium caused by a situation in which (1) one believes one knows the ethically ideal action to take and (2) that one cannot carry out that action because of (3) institutionalized obstacles such as lack of time, lack of supervisory support, medical power, institutional policy, or legal limits’.	Psychological effects Having formed a moral belief Desired outcome not achieved Institutional constraint
5.	Corley et al.^18^ (p. 250)	‘Jameton^1^ defines as moral distress: the painful psychological disequilibrium that results from recognizing the ethically appropriate action, yet not taking it, because of such obstacles as lack of time, supervisory reluctance, an inhibiting medical power structure, institution policy, or legal considerations’.	Psychological effects Having formed a moral belief Desired outcome not achieved Institutional constraint
6.	Corley^47^ (p. 643)	‘Moral distress is the psychological disequilibrium, negative feeling state, and suffering experienced when nurses make a moral decision and then either do not or feel that they cannot follow through with the chosen action because of institutional constraints’.	Psychological effects Having made a moral judgement Desired outcome not achieved Institutional constraints
7.	Hanna^8^	‘An ‘umbrella category’ that could include the experience of anguish or suffering associated with facing a moral dilemma, moral uncertainty as well as certainty accompanied by constraint’.	Overarching term Psychological effects Similar to a moral dilemma, moral uncertainty and moral certainty Constraint
8.	Kälvemark et al.^19^	‘Traditional negative stress symptoms that occur due to situations that involve ethical dimensions and where the health care provider feels she/he is not able to preserve all interests and values at stake’.	Psychological effects An ethical problem Compromised values
9.	Peter and Liaschenko^20^	‘if moral agency is defined as the capacity to recognize, deliberate/reflect on, and act on moral responsibilities, in order to experience moral distress, an agent is required to possess at least some autonomy in recognizing and reflecting upon moral concerns. Yet on the other hand, an agent’s autonomy must be at least somewhat constrained in acting upon the very moral responsibilities he/she understands him/herself to have. This apparently irresolvable contradiction is moral distress’.	Moral agency/moral autonomy Constraint on moral agency/moral autonomy
10.	Corley et al.^21^	‘Jameton,^1^ who defined it as painful feelings and/or the psychological disequilibrium that occurs when nurses are conscious of the morally appropriate action a situation requires but cannot carry out that action because of institutionalized obstacles’.	Psychological effects Being aware of a moral belief Desired outcome not achieved Institutional constraints
11.	American Association of Critical Care Nurses^42^ (p. 1)	‘Moral distress occurs when: You know the ethically appropriate action to take, but are unable to act upon it. You act in a manner contrary to your personal and professional values, which undermines your integrity and authenticity’.	Having made a moral judgement Constraint Moral integrity compromised
12.	Nathaniel^48^ (p. 421)	‘Moral distress is pain affecting the mind, the body, or relationships that results from a patient care situation in which the nurse is aware of a moral problem, acknowledges moral responsibility, and makes a moral judgment about the correct action, yet, as a result of real or perceived constraints, participates, either by act or omission, in a manner he or she perceives to be wrong’.	Psychological effects Physical effects Aware of a moral problem Acknowledges moral responsibility Makes a moral judgement Constraint or perceived constraint Desired outcome is not achieved
13.	Canadian Nurses Association^49^ (p. 6)	‘Ethical (or moral) distress arises in situations where nurses know or believe they know the right thing to do, but for various reasons (including fear or circumstances beyond their control) do not or cannot take the right action or prevent a particular harm. When values and commitments are compromised in this way, nurses’ identity and integrity as moral agents are affected as they feel moral distress’.	Having formed a moral judgement or a moral belief Constraints Values compromised Commitments compromised Moral identity compromised
14.	McCarthy and Deady^9^	‘…an umbrella concept that captures the range of experiences of individual who are morally constrained. Generally speaking, when individuals make moral judgments about the right course of action to take in a situation, and they are unable to carry it out, they may experience moral distress. In short, they know what is the right thing to do, but they are unable to do it; or they do what they believe is the wrong thing’.	Overarching term Constraint Having made a moral judgement Desired outcome is not achieved
15.	McCarthy^50^(p. 1)	‘Moral distress is an umbrella concept that describes the psychological, emotional and physiological suffering that may be experienced when we act in ways that are inconsistent with deeply held ethical values, principles or moral commitments’.	Psychological effects Physiological suffering Compromised ethical values, principles or moral commitments
16.	Jameton^22^	‘Moral distress- a common experience in complex societies- arises when individuals have clear moral judgments about societal practices, but have difficulty in finding a venue in which to express concerns’.	Having made a moral judgement Affects society as a whole Unable to express concerns Desired outcome not achieved
17.	Hamric and Wocial, personal communication, October 24, 2013 in Hamric^43^	‘Moral distress occurs when an individual’s moral integrity is seriously compromised, either because one feels unable to act in accordance with core values and obligations, or attempted actions fail to achieve the desired outcome’.	Moral integrity compromised Desired outcome not achieved, despite efforts.
18.	Barlem and Ramos.^24^	‘…the feeling of powerlessness experienced during power games in the micro-spaces of action, which lead the subject to a chain of events that impels him or her to accept imposed individualities, have his or her resistances reduced and few possibilities of moral action; this obstructs the process of moral deliberation, compromises advocacy and moral sensitivity, which results in ethical, political and advocational inexpressivity and a series of physical, psychical and behavioural manifestations’.	Constraint on moral action Constraint on moral deliberation Constraint on one’s ability to advocate Reduction of moral sensitivity Feelings of powerlessness Physical, psychological and behavioural effects
19.	Fourie^25^	‘Moral distress is a psychological response to morally challenging situations such as those of moral constraint or moral conflict, or both’.	Psychological effects Morally challenging situation
20.	Campbell et al.^26^	‘One or more negative self-directed emotions or attitudes that arise in response to one’s perceived involvement in a situation that one perceives to be morally undesirable’.	Self-directed psychological effects Morally undesirable situation

We did not follow these seven steps in a linear fashion but used an iterative process, moving between each stage in the direction that made sense of the data.^13^

## Findings

From the 34 included papers, 20 key definitions were identified (Table 3). Rather than discuss each definition in depth, our focus is on exploring the necessary/sufficient conditions that make up each definition, and we discuss these thematically.

## Moral judgements

Jameton’s^1^ definition stipulates moral judgement and constraint as necessary and sufficient conditions for MD and differentiates MD from ‘moral dilemmas’ and ‘moral uncertainty’. According to Jameton,^16^ the nurses he encountered were describing as ‘dilemmas’ instances where they had made a moral judgement but were unable to act upon it, and this is what caused their MD. Similarly, Peter et al.^27^ found that although uncertainty pervaded nurses’ narratives of aggressive care, they only experienced what they called MD, in situations where they ‘knew’ the right thing but were constrained. The subsequent narrative surrounding MD focused on the complexities of negotiating their moral judgements in the presence of constraints.^27^ To make sense of this definition, we must be clear about what it is to have made a moral judgement and to be constrained. There is little ambiguity about how to understand ‘constraint’ – it is a barrier to acting as one would want. There is, however, ambiguity about what a ‘moral judgement’ is. Terminology in the literature ranges from a ‘moral judgement’, ‘moral decision’, ‘moral belief’ or an ‘awareness’ (see definitions 1, 2, 3, 5, 6, and 11 in Table 3). Considering these terms can mean different things, when used interchangeably we are faced with the problem of whether they are being used, perhaps erroneously, as a synonym for ‘moral judgement’ understood consistently, or if a different meaning is intended. Whether we can, therefore, accept ‘moral judgement’ as a necessary condition of MD will depend on what we mean when we say ‘moral judgement’.

Fourie^25^ critiqued Jameton’s^1,16^ definitions of MD on the basis that they are unacceptably narrow. If moral judgement is a necessary condition of MD, then it cannot occur in situations characterized by indecision, such as moral dilemmas or uncertainty. On this basis, many definitions in Table 3 could be critiqued as too narrow (with a few exceptions, namely, Kälvermark et al.,^19^ Fourie^25^ and Campbell et al.^26^). Fourie’s criticism is that while moral judgement might be a sufficient condition for MD (when combined with constraint), it ought not be thought necessary because MD can be experienced in the absence of a moral judgement, for example, when one is faced with a moral dilemma and feels uncertain about what judgement should be made.

## The psychological and physical effects of MD

Jameton’s^1^ definition can also be viewed as too narrow due to the exclusive focus on causal conditions. On his account, there is no necessary affective component to MD, and MD could occur without anyone actually feeling distressed, as all that is needed is a moral judgement that cannot be acted upon. We might reasonably assume that the feeling of distress is implicit on Jameton’s definition, but Wilkinson^5^ was the first to explicitly incorporate the psychological effects of MD into a definition. After carrying out interviews with 24 nurses, Wilkinson^5^ described seven ‘indicators’ that were perceived to contribute to, or were influenced by, MD. Wilkinson^5^ concluded that MD produced feelings of anger, frustration and guilt, which were produced in response to one’s moral decision being thwarted. Wilkinson^28^ argued that Jameton’s definition failed to refer to the effects (psychological distress) of MD and only indicated the cause (judgement + constraint) and so incorporated both cause and effect into her definition of MD (Table 3, definition 2). Since Wilkinson, it seems to be taken for granted that MD has both a causal and affective component, requiring a particular cause and particular response.

What that response is, and whether any are necessary and/or sufficient, is unclear. The qualitative literature captures a broad range of likely psychological and physical effects of MD. In Wiegand and Funk,^29^ nurses discussed feeling frustration, anger, sadness, psychological/physical exhaustion, helplessness, distress and depression. While Hanna^8^ described the physical effects of MD as sleeplessness, nausea, migraines, gastrointestinal upset, tearfulness and physical exhaustion.

## Moral dilemmas and uncertainty

Fourie^25^ argued that Jameton^16^ used the terms ‘moral dilemma’ and ‘moral conflict’ interchangeably, implying a commonsense understanding of ‘moral dilemma’, and that being where we are faced with a difficult moral decision but, with enough thought, it is possible to identify the morally correct action. Conversely, the standard philosophical view of moral dilemmas is that a dilemma occurs where there are two competing and equally strong obligations that cannot both be met.^25^ This leads to a further narrowing of Jameton’s definition, as it suggests that no instances of uncertainty (whether a moral conflict or a genuine dilemma) can be a sufficient condition of MD.

Some empirical researchers, such as Kälvemark et al.,^19^ found that distress occurred during moral dilemmas and uncertainty, when healthcare professionals (HCPs) were uncertain about the right course of action, and called this MD. This resulted in a definition (definition 8, Table 3) of MD that disassociated MD from moral judgement and turned it into a negative psychological response to a perceived inability to act in the moral interests of all stakeholders and uncertainty about whose interests ought to be prioritized. This account adds moral uncertainty and experiencing dilemma as a sufficient condition for MD, but does not rule out constraint. Inability to act in all stakeholders’ moral interests could flow from both failing to make a decision (uncertainty, genuine dilemma) and from the inability to act (constraint).

More recently, Campbell et al.^26^ argued that six causes of MD (moral uncertainty, mild distress, delayed distress, moral dilemma, bad moral luck and distress by association) fell outside Jameton’s^1^ definition and argued this should motivate a broader understanding of MD that could accommodate all of these causes as sufficient (definition 20, Table 3).

These responses to Jameton^1^ are examples of typical responses that challenge the idea of moral judgement and/or constraint being a necessary condition of MD and seek instead to place it as one of the many causal conditions that may be sufficient when combined with others. Some 30 years after publishing his original definition, Jameton,^22^ however, appears to have gone further, rejecting moral judgement as even a sufficient condition and making moral uncertainty a necessary condition. In Jameton’s^22^ words:

…[m]oral distress expresses a decision point, a moment of emotive immobility, where ambivalence needs to be resolved toward a choice. Once the choice is made and action is undertaken, the psychological elements of distress tend to diminish.

Here, Jameton^22^ implies that MD occurs exactly when one is forced to choose between two similarly weighted actions (a moral dilemma or difficult decision), where different obligations conflict and the correct course of action is uncertain. Psychological distress follows from the inability to decide, receding once a decision is made.

Jameton^22^ still appears to imply that there is a correct course of action and once it has been identified, the decision-maker will no longer feel distressed. Against this, Weinberg^30^ argues that MD can occur when there is no ‘correct’ course of action identified. It seems just as plausible that MD could follow from an unsatisfactory decision as from uncertainty and inaction.

## Constraints as causes of MD

Jameton’s^1^ definition framed MD as a purely occupational issue, arising because of institutional barriers or constraints.^8,31^ This resulted in decades of research that assumed constraint to be a necessary condition of MD, exploring the nature and kind of constraints that caused MD, and using the presence of constraint, and responses to it, as a way of measuring MD and its prevalence.

### The Moral Distress Scale

Corley^17^ developed the first Moral Distress Scale (MDS), underpinned by definition 4 (Table 3), and a revised scale in 2005, underpinned by definition 10 (Table 3). The MDS lists possible scenarios where a nurse is constrained from carrying out their preferred moral action, asking respondents to score the frequency and intensity of MD. The MDS was further revised by Hamric et al.^32^ who developed the Moral Distress Scale–Revised (MDS-R). These scales have been frequently modified by individual researchers from different countries who have, arguably, equivocated conceptions of MD while using an instrument constructed using a specific conception of MD, giving us reason to doubt the internal consistency of their studies. For example, Browning^33^ used and modified the MDS, a measure of constraint, and yet stated that MD occurred during moral dilemmas, defining MD as ‘discomfort or internal conflict related to ethical dilemmas encountered in nursing practice when constraints prevented the nurse from following the course of action believed to be right’. Hamric^7^ highlights how ‘valid measures require a tight linkage between the concept and the items developed for the measure. It is clear that, at present, multiple measures exist which measure different concepts’. This not only raises questions about internal consistency but it also creates difficulties when conducting meta-analysis; even when studies purportedly use the same measure, they seem to be discussing different concepts and so cannot be readily compared.

### Ethical climate

Further examination of constraint on action led to researchers exploring the relationship between constraint on actions and the ethical climate of institutions. Again, Corley et al.^21^ led the way with quantitative exploration using a revised MDS and an Ethical Environment Questionnaire (EEQ).^34^ Subsequently, others have found positive perceptions of ethical climate to be associated with lower MD scores.^6,32,35,36^ Evidencing links between MD and institutional ethical climate is useful for developing ways to reduce MD. Musto and Rodney^37^ argue, however, that they are overly simplistic, mapping correlations only, and do not illuminate the complex interplay between an individual’s moral agency, the institution’s interests and resulting MD.

Possibly, with this kind of critique in mind, Peter and Liaschenko^20^ suggest reframing MD in terms of having moral agency that one is unable to act on, thereby being unable to fulfil one’s perceived moral responsibilities (definition 9, Table 3). Adopting a feminist ethical framework, Peter and Liaschenko^38^ explore the personal or perceived constraints that cause MD. They suggest that MD is a response to constraints on nurses’ moral identities, responsibilities and relationships rather than a response to specific external causes. They emphasize the social connectedness of ethics and the belief that moral knowledge is born out of shared moral experiences. They argue that institutions often create constraints on nurses’ moral identities (rather than on discrete actions), restricting their ability to act as autonomous moral agents and so preventing them from acting in accordance with their core values and professional responsibilities. For these authors, the violation of one’s moral agency, rather than constraint on action, is the necessary and sufficient condition for MD. Constraint on action may cause this violation, but it is not the only possible cause. If this is the case, then qualitative rather than quantitative methods may be more suitable to gather the rich data required to explore violations of moral identity and moral agency.

### Internal constraints

Epstein and Hamric^39^ suggest that MD can also be caused by internal constraints on one’s moral actions. Internal or personal constraints are regarded as self-doubt, lack of assertiveness, socialization to follow orders, perceived powerlessness and lack of understanding. Internal constraints have received very little attention within the quantitative literature, with limited qualitative exploration. It is likely that this lack of exploration is due to MD being predominantly conceptualized as arising from external constraint on action. Barlem and Ramos^24^ theorize that it is the power play in various ‘micro-spaces’ which can create internal constraints which impede one’s ability to deliberate about moral issues.

### Epistemic injustice

One suggested form of constraint on the moral agency of nurses identified by Reed and Rishel^40^ is epistemic injustice. They argue that MD occurs because often nurses aren’t informed of treatment decisions and their views often not incorporated into decision-making or interdisciplinary discussions, creating ‘epistemic inequality’ in the workplace. Insofar as nurses (1) have to convey and enact decisions made by others, (2) are in a position of epistemic uncertainty and (3) have not been a part of the process, they are acting as mouthpieces for others rather than as autonomous moral agents. Reed and Rishel argue that this is an epistemic injustice, ‘a wrong done to someone specifically in their capacity as a knower’. This is based upon the work of Fricker,^41^ who argues there are two kinds of epistemic injustice: testimonial and hermeneutical.

In testimonial injustice, the speaker is discredited because of the listener’s prejudice. This can occur in nurse–physician interactions in the context of end-of-life decisions, where nurses’ are undermined in their capacity as knowers, experience epistemic injustice and, consequently, MD.^40^ Hermeneutical injustice occurs when a nurse’s professional opinion is undermined and ignored. Reed and Rishel^40^ argue this is an example of discrimination against one’s social identity; nurses are below doctors in the medical hierarchy. They argue that ‘this hinders development of intellectual courage, selfhood, and well-being, as well as impoverishes disciplinary knowledge overall’.^40^ Arguably, on this account, the MD an agent experiences as a result of either kind of epistemic injustice flows from the agent being wronged by a failure to respect her as a moral agent. This conception of MD is also supported by Peter el al.^27^ who provide quotes from nurses who have experienced both forms of epistemic injustice. It is not clear, however, to what extent epistemic injustice could be considered a necessary condition of MD or whether it is simply another example of a way in which one’s moral agency can be constrained.

## Threat to moral integrity

In 2006, the American Association of Critical Care Nurses (AACN)^42^ released a position statement claiming that the inability to act upon personal and professional values undermines integrity and authenticity, and this is core to the experience of MD (definition 11, Table 3). This distinction between professional and personal values does not feature in previously suggested definitions of MD, despite some discussion in the literature.^8,23^

Although integrity has not received the same attention as constraint, it has not been entirely ignored. Recently, Hamric^43^ and Wocial also suggested that integrity is central to MD (definition 17, Table 3) and that compromised moral integrity causes an emotional distress response, such as avoidance, frustration and anger. Thomas and McCullough^44^ also developed an account that draws on moral integrity, arguing that MD could be divided into six philosophical categories: challenges to, threats to, and violations of professional integrity; and challenges to, threats to, and violations of personal integrity. They argued that these categories place different values under threat and thereby cause different degrees of MD. These accounts are consistent with Wilkinson’s^5^ cause and effect model, and frame MD in terms of necessary cause (threat to moral integrity) and necessary effect (psychological distress). Arguably, however, the use of moral integrity does not bring any clarification, as it is itself an ambiguous concept, that may include all the sufficient causes hereto discussed.

Thomas and McCullough^44^ used Beauchamp and Childress^45^ definition of moral integrity, characterizing it as ‘soundness, reliability, wholeness, and integration of moral character’, and ‘objectivity, impartiality, and fidelity in adherence to moral norms’. Hardingham,^46^ for whom moral integrity is a necessary condition of MD, adopted an interpretation from the political philosopher Larry May, where moral integrity refers to ‘a wholeness in the relationship between our actions and our values and beliefs…about a certain conception of our self as being a consistent whole’. Like MD, moral integrity can be understood in a variety of ways and is contested. As such, introducing the concept of moral integrity in order to bring about conceptual clarification of MD does not work – it merely defers the problem.

## Discussion

The review has highlighted, and unpicked, the many different conceptual foundations of MD in the literature. ‘Narrow’ conceptions of MD sprung from Jameton’s^1,16^ definition which considered both moral judgement and constraint necessary and sufficient conditions of MD. One of the main difficulties with Jameton’s original account was the condition of ‘moral judgement’ being ambiguous, leading to different understandings of MD. Across a range of accounts that purported to be consistent with Jameton, ‘moral judgement’ seems to be used inconsistently, referring (in different accounts) to apparently different cognitive states of varying epistemic strength. For example, to have a belief ‘that x’ seems to be saying something quite different, and stronger, to having an ‘awareness of x’; and yet, all these cognitive states, collectively referred to as ‘moral judgement’, are seen as equivocal to the basic state of an agent having made a decision regarding the right thing to do. This might be argued to be simply an inconsequential difference of expression rather than meaning, but it is problematic because consistent and unambiguous language is vital when trying to understand a complex concept. Furthermore, in the context of moral philosophy, the difference between labelling something as a belief, a judgement or an awareness can be significant and would alter the conditions for MD. On a more practical level, questionnaires designed to measure MD are reliant on participants and researchers having the same understanding of ‘moral judgement’ for their effectiveness. Risk of conceptual conflation and equivocation is high.

Fourie^25^ also highlighted that Jameton’s^16^ interchangeable use of the terms ‘moral dilemma’ and ‘moral conflict’ implied that he adopted a commonsense notion of moral dilemmas, aligning dilemma with moral conflict. Accepting this interpretation leads to a conception of MD that excludes experiences associated with internal conflict, dilemma or uncertainty, and this seems to conflict with empirical accounts of MD. One response may be that people who give these accounts are mistaken about what MD is because they have not met the necessary and sufficient conditions of judgement and constraint. Alternatively, we could accept those accounts and expand Jameton’s narrow definition to accommodate them. This requires us to explain MD in terms of a distress response to a range of possible causes, including moral conflict and uncertainty.

The idea of constraint as a sufficient condition for MD has been consistently used and the notion that it is a necessary condition has been perpetuated, in part, through the use of quantitative measures of MD, such as the MDS and MDS-R. These presuppose constraint as the cause of MD and therefore only measure MD as a phenomenon arising from constraint. The use of a scale both ‘pre-codes’ and interprets given situations as causing MD and therefore primes the participant to accept this account of MD as a ‘reality’.^18^

These problems with narrow conceptions of MD led to broader definitions, which initially included the psychological (and physical) effects.^5^ However, Fourie^25^ has argued that stipulating both specific cause (constraint) and effect (psychological effects) simply creates a ‘compound’ definition that is also too narrow and overlooks other causal conditions of MD, such as moral conflicts, dilemmas or uncertainty. This criticism, however, appears to be about the exclusive nature of Jameton’s account (limited as it is to just one cause), rather than its compound nature, and could be dealt with by extending the range of causes, which is what most subsequent accounts of MD have tried to do.

The psychological distress component of MD, however, seems to have become a necessary condition of MD. Indeed, if we appeal to a commonsense understanding of the term ‘moral distress’, it seems obvious that any distress causally associated with a ‘moral event’, such as a moral dilemma or moral uncertainty is, ipso facto, MD. Although commonsensical, it does not necessarily clarify anything, and the problem remains of defining what a ‘moral event’ is and of determining what the causal association between the ‘moral event’ and the distress looks like.

Arguably, psychological distress is a necessary condition of MD but not a sufficient one. A person may experience psychological distress linked to life events but to be properly labelled MD, it seems necessary that the distress is directly causally related to a ‘moral event’. This would make the combination of (1) the experience of a moral event, (2) the experience of ‘psychological distress’ and (3) a direct causal relation between (1) and (2) necessary and sufficient conditions for MD.

## Conclusion

Research suggests that MD negatively affects nurses. In order to support nurses through their experiences of MD, we need to understand both the phenomenon and the context in which it occurs. We have shown that there is still little agreement about the conditions that cause MD and therefore there is doubt about what MD is and when and how it occurs. By analysing the key definitions of MD and suggesting which conditions could be regarded as necessary and/or sufficient, we have, however, contributed to definitional and conceptual clarity that is required for us to increase our understanding of MD and shape our responses to it.

## Supplemental material

Supplemental Material, NEJ_Supplementary_Material_(Table_4) - What is ‘moral distress’? A narrative synthesis of the literature Supplemental Material, NEJ_Supplementary_Material_(Table_4) for What is ‘moral distress’? A narrative synthesis of the literature by Georgina Morley, Jonathan Ives, Caroline Bradbury-Jones and Fiona Irvine in Nursing Ethics
