# Supplementary material for: What is ‘moral distress’? A narrative synthesis of the literature
Source: Nurs Ethics. 2017 Oct 8;26(3):646–62. doi: 10.1177/0969733017724354 (PMC6506903; doi:10.1177/0969733017724354)
Supplement: Supplemental Material, NEJ_Supplementary_Material_(Table_4) - What is ‘moral distress’? A narrative synthesis of the literature [file NEJ_Supplementary_Material_(Table_4).pdf]

Table 4: Papers included in the narrative synthesis (alphabetical order)

Reference, Setting, Study Design	Study Aim, Sample	Method	Key Findings/ Key Arguments
Allen et al.,[1] USA, quantitative	To measure moral distress among healthcare professionals (HCPs) working in adult and paediatric settings, to explore differences between professions and demographic factors in relation to levels of MD. 1 healthcare system (4 adult acute, 2 district hospitals, 1 children's community hospital) Participants *(n=323)* recruited: RNs *(n=207:* 194 adult, 13 paediatric); advanced registered nurse practitioners (ARNPs) *(n=7)*; physicians *(n=62:* 51 adult, 11 paediatric); social worker/ case manager *(n=27);* respiratory therapists *(n=20).*	Moral Distress Scale-Revised (MDS-R) from Hamric et al.[2], 0-4 scale, 6 parallel versions for RNs, Drs, other HCPs & adult & paediatric settings. Internal consistency-Cronbach α ranged from 0.88-0.95.	MD defined similarly to Jameton[3]. Overall high levels of MD. ARNPs-highest mean MD scores, followed by respiratory therapists (RT). RNs & RTs -same 2 highest scoring MD items. Higher MD scores in adult setting compared to paediatric *($p=0.007$).*Higher MD scores associated to those considering/ had considered leaving/ left a previous position due to MD *($p<0.001$).*
Astbury et al.,[4] UK, theoretical	To determine whether there is scope to study moral distress in pharmacists.	Literature review, not systematic.	MD defined according to Jameton[3]. Suggest MD occurs when individuals are prevented from exercising their moral agency and act in discordance with their moral judgements & values. Suggest that because pharmacists are evolving their role to include responsibility for optimal drug-therapy & patient focused care- there are more opportunities for ethical & moral problems to arise.
Barlem & Ramos,[5] Brazil, theoretical	To formulate a theoretical model of moral distress to clarify the concept and to broaden the definition.	Theoretical.	New definition of MD suggested in which MD arises due to constraints on moral action, moral deliberation, moral sensitivity and advocacy, MD is connected to feelings of powerlessness and causes "ethical, political and advocational inexpressivity" and "physical, psychical and behavioural manifestations."
Campbell et al.,[6] USA, theoretical	To motivate a broader understanding of moral distress and argue for a broader definition.	Theoretical, hypothetical case studies.	6 cases of distress that can be understood as MD: 1. Moral uncertainty. 2. Mild distress. 3. Delayed distress. 4. Moral dilemma. 5. Bad moral luck. 6. Distress by association. New suggested definition of MD: "one or more negative self-directed emotions or attitudes that arise in response to one's perceived involvement in a situation that one perceives to be morally undesirable".

Corley, [7] USA, quantitative	To measure levels of nurse moral distress using a newly developed instrument- the Moral Distress Scale (MDS). Members of the mid-Atlantic, American Association of Critical-Care Nurses *(n=111).*	32-item MDS, 1-7 scale, based on Jameton's [3] & Wilkinson's [8] conception of MD. Scale reviewed by 3 nursing ethics experts for content validity & test-retest reliability r=0.86 (*p<0.01*), Cronbachs α = 0.93.	MD defined as "painful feelings &/or psychological disequilibrium caused by a situation in which (1) one believes one knows the ethically ideal action to take & (2) that one cannot carry out that action because of (3) institutionalized obstacles such as lack of time, lack of supervisory support, medical power, institutional policy, or legal limits" (p.280). Mean MD scores were below the mid-point suggesting low levels of MD. Most frequent: 'Initiate dramatic life-saving actions when I think it only prolongs death' (M=5.9; SD= 1.37). 'Following the family's wishes to continue life support even though it is not in the best interest of the patient' (M=5.5; SD= 1.27). Greatest intensity of MD: Giving medication intravenously to a patient who had refused to take it orally' and 'Giving only haemodynamic stabilizing medication intravenously during a Code Blue with no compression or intubation.' (no mean or SD provided). No significant findings between MD score & demographic factors.
Corley et al., [9] USA, quantitative	To explore relationships between MD intensity & frequency, correlations to ethical work environment & demographic characteristics of nurses. RNs *(n=106)* from 2 large medical centres, medical & surgical units. 62% response rate (RR).	Correlational study. Revised version of the Moral Distress Scale (MDS)- originally 32-items, now a 38-item questionnaire (0-6 scale) (problems identified in previous studies added: pain management, managed care & incompetent HCP's). Measures MD frequency & intensity, Tool reviewed & revised by experts. Cronbach α for the revised MDS intensity scale=0.98 & MDS frequency scale= 0.90. Ethical Environment Questionnaire (EEQ) developed by McDaniel [10] - 20-item questionnaire with 5-point style Likert scales. Cronbach α= 0.93.	Elaborates but references Jameton's [3] definition: "painful feelings and/or the psychological disequilibrium that occurs when nurses are conscious of the morally appropriate action a situation requires but cannot carry out that action because of institutionalized obstacles."(p.382) Lower perception of ethical climate correlated with more MD intensity & frequency *(p=0.01).* Lowest intensity item: 'give medication intravenously during a code with no compressions or intubation.' Highest frequency: not stated. Lowest frequency: 'respond to patient's request for suicide assistance when the patient has a poor prognosis'. African Americans had higher MD scores- authors question whether less empowered.
Dzeng et al.,[11] USA, qualitative	MD emerged as a major theme when exploring physician & trainee views on resuscitation orders & end of life- this paper was produced in response to this finding. Qualified physicians carrying out further medical training *(n=22)* from 3 sites, participants selected based on a range of years of experience *(1-6 years).* Participants excluded if they had not attended medical school and residency (3-7 years, or more of continuing medical training in the US).	Qualitative in-depth interviews with an interview guide. 2 interviews conducted via Skype, all other conducted in person. Lasted 45- 120 mins, audiotaped, transcribed verbatim. Data collected until theoretical data saturation achieved.<br>"Moral distress occurs when individuals believe they are unable to act in accordance with their ethical beliefs due to hierarchical or institutional constraints"- reference to & loosely based on Jameton's[3] 1984 definition of MD.	Key themes with direct quotations to support: 1. Perceived futile end of life care-'torture'& causing 'suffering' 2. Practitioner suffering & emotional angst- due to providing perceived futile care. 3. Feelings of powerlessness- unable to terminate futile care. 4. Hierarchy- attributed their powerlessness to the hierarchy, trainees unable to question superiors.<br>5. Dehumanization & rationalization- distancing themselves from patients- describing patients as a 'body' not a 'person'. 6. Coping strategies- institutions that encourage conversations to reflect & share emotions, notify healthcare professionals involved about deaths.

Epstein & Hamric, [12] USA, theoretical	To propose a preliminary model- the "crescendo effect" -which describes the relationship between moral distress and moral residue.	Theoretical	MD= initial distress- conflict of values. Moral residue= reactive distress- the psychological effects. MD is not the psychological effects- it is a purely ethical phenomenon. Psychological distress such as under-staffing does not violate core values as one can act on their ethical obligations still. Associating MD with psychological distress minimizes the experience to a mere psychological phenomenon, rather than ethical. The crescendo effect arises due to moral residue, which serves as a new baseline each time there is a new morally distressing situation- both MD and moral residue accumulate to a crescendo, which can cause HCPs to leave their profession.
Fourie, [13] Switzerland, theoretical	To clarify the concept of MD.	Conceptual analysis with focus on Jameton's definition of MD from his 1993 paper.	Argue that Jameton's definition implies MD & moral dilemmas are mutually exclusive. Jameton fails to differentiate between a moral dilemma (in the strict, philosophical sense) and moral conflict, thereby amounting to the claim that MD & moral conflicts are mutually exclusive. Jameton's definition is compound (a 'narrow' definition) because the specific cause & response are built into the definition, making moral constraint a necessary condition of MD. Situations of moral uncertainty, moral dilemma & moral conflict are ruled out as causing MD. New definition suggested.
Hamric et al.,[2] USA, quantitative	To develop and test a revised version of Corley's MDS-the Moral Distress Scale-Revised (MDS-R). Aim of the MDS-R is to include more root causes of MD, expand its use outside of intensive care unit (ICU) & make it appropriate for other HCP's. RNs *(n=169:* 131 adult, 38 paediatric*)* 48% RR. Physicians *(n=37:* 12 fellows, 8 residents, 17 attending physicians*)* 60% RR. From 8 ICUs: 6 adult, 2 paediatric from one academic medical centre in southeastern US.	MDS-R - MDS updated, shortened, re-worded, applicability broadened & space for 2 free-text responses- in consultation with Corley. 6 parallel versions developed for nurses, physicians, other HCP's in adult & paediatric settings.0-4 Likert scale, measures frequency & intensity, 21-items. Content validity performed with authors & doctoral prepared colleagues in nursing ethics. Cronbach α = 0.67 for physician scale & 0.89 nurses. Shortened version of Olson's HECS Cronbach α = 0.77 for physician scale & 0.87 nurses.	Pre-defined MD at start of the survey- definition not provided. Drs (Mean (M) 62.58; Standard Deviation (SD) 21.92) had significantly lower MD scores than RNs (M 91.53; SD 44.24) *(p<0.0001)* but similar mean scores in perceptions of ethical climate- RN (M 48.54; SD 8.27) Dr (M 52.13; SD 6.36). RNs & Drs agreed on 6/7 top situations to cause MD. Top 2 greatest frequency: 'Follow the family's wishes to continue life support even though I believe it is not in the best interest of the patient' & 'Initiate extensive lifesaving actions when I think they only prolong death.'
Hamric,[14] USA, theoretical/ case study	To analyse a case study that caused moral distress, identify themes and suggest strategies to reduce MD.	Case based discussion developed from the authors previous experiences working with morally distressed clinicians.	MD consists of having one's moral values/ obligations compromised. Moral residue is lasting & powerful because action/inaction has resulted in threatening core beliefs & values that help define oneself. MD is subjective because everyone holds different beliefs. Presents a case of futile care & the effects on the healthcare team. MD permeates across 3 levels- individual, team/unit, institution/ organization. Emphasizes the importance of team communication, getting to the roots of the MD. New definition suggested.

Hanna, [15] USA, theoretical	To provide an overview of research related to moral distress.	Theoretical	Due to Jameton's[3] 1984 definition- MD was explored as an occupational issue. However nurses more often speak about the psychological effects of MD- this reveals a mismatch between the experience of MD and his definition. Raises the issue of role morality and questions whether it is even possible to separate oneself in such a way. Hanna identifies 4 themes in current research (of note, the studies she looked at were mostly unpublished PhD & MSc dissertations). 1. Anguish or interior suffering. 2. Role Morality. 3. Truth telling 4. Conflict associated with early insights.
Hanna, [16] USA, qualitative	To discover the essence, properties & full-content domain of the concept of MD & to create a universal definition. Variation sampling to recruit RNs *(n=10)* who have assisted with legal, elective abortions. Screened participants prior to recruitment to ensure all had assisted with elective abortions & had experienced MD related to the procedure- although it is not stated how they pre- screened. Interviewed one nurse who had not experienced MD for comparison.	Semi-structured interview-guided by phenomenology & aspects of the Roy Adaptation Model (RAM) model of nursing- this work emphasizes the holistic unity of human persons. Conceptual basis for the study is verativity- the view humans are in a relationship with a loving Creator & universal truths are discoverable.	MD defined as "an employer/employee conflict, whereby the employee knew the right thing to do, but was prevented by the employer from carrying it out." (p.96)- attributes this to Jameton 1984. 5 properties of MD are identified: 1.Perception. 2. Pain.<br>3. Valuing. 4. Altered participation. 5. Perspective. 3 types of MD identified: 1.Shocked. 2. Muted. 3. Suppressed (persistent).
Hardingham,[17] Canada, theoretical	To discuss the concepts of MD, moral integrity & moral residue in relation to nursing practice in Canada and to understand how ethical nursing practice can be strengthened.	Theoretical.	Moral integrity is necessary to a moral life and is relational in nature. When integrity is threatened MD & moral residue occur. Introduced philosopher (Larry May) interpretation of integrity- as coming to maturation through reflection, developing a critical coherence which is often done through socialization in a professional group. Advocacy can harm nurses because the orgnanisation have the power, not individual RNs- institutional barriers cause MD. Nurses are forced to go against their moral integrity & to change this there needs to be a change in the practice environment, organizational culture and education of nurses. Suggests building a moral community as an environment to practice ethically.
Jameton,[3] USA, theoretical	To describe the ethical issues in nursing practice.	Theoretical.	First introduction of moral distress to nursing practice. MD defined as occurring "when one knows the right thing to do, but institutional constraints make it nearly impossible to pursue the right course of action" (p.6). Distinguishes between MD, moral uncertainty and moral dilemmas- stating that moral & ethical problems in healthcare are of these 3 types.

Jameton, [18] USA, theoretical	No aim stated.	Theoretical.	Distinguishes between initial & reactive distress. Jameton redefines MD, argues his 1984 definition is initial MD and Wilkinson's[8] definition is reactive MD. Initial distress involves feelings of frustration, anger & anxiety when faced with institutional obstacles & disputes with others regarding values, and reactive distress occurs when one fails to act upon their initial distress. Many causes of MD are rooted in questions of moral responsibility- nurses have many options, but it is difficult to know which option to take- Jameton argues this engenders moral dilemmas, it is questions of moral responsibility, rather than moral dilemma that cause MD.
Jameton, [19] USA, theoretical	To discuss and extend the concept of MD beyond the healthcare setting and apply it to climate change.	Conceptual analysis of moral distress.	Discusses evolution of concept- argue it represented the issues of power & inequality that nurses faced. Feminist ethics only encouraged passivity & regret, later replaced with assertiveness & the call to speak out on behalf of their profession. As other professions with their own ethical codes developed, it became evident MD wasn't just experienced by nurses but almost anyone working in an organization. Climate change is an increasing ethical problem and those concerned about it experience MD- there is very little control over environmental ethics that can be exercised at the bedside. Bioethicists ought to move beyond the MD of clinical matters to global ethics.
Johnstone & Hutchinson, [20] USA, theoretical	To discuss the nature of MD and discuss the quality and safety of moral decision-making, moral conduct and moral outcomes in nursing and healthcare	Theoretical- using empirical research as evidence.	MD is conceptually flawed, lacks an empirically robust foundation which although extensive, is methodologically weak & disparate. The hypothesis that MD occurs when nurses know the right thing is concerning for 3 reasons: 1. It assumes nurses know the right thing uncritically. 2. It is apologist & perpetuates the notion of nurses as powerlessness. 3. It is question- begging- it takes for granted that MD is a "bona fide state" causally related to 1 and 2.
Kälvemark et al., [21] Sweden, qualitative	To explore which situations involve ethical dilemmas and whether they cause moral distress. HCPs (nurses, doctors, auxiliary nurses, medical secretaries and pharmacy staff).	Focus groups in 1 cardiology, 1 heamatology, 1 pharmacy department in Stockholm. 5-7 HCPs in each focus group representing the different professions. 2 researchers present- 1 moderator, 1 note-taker. Sessions last from 1.5-2 hours, recorded & transcribed. The authors do not describe their data analysis techniques.	MD redefined as: "Traditional negative stress symptoms, such as feelings of frustration, anger, and anxiety, which might lead to depressions, nightmares, headaches and feelings of worthlessness, that occur due to a conviction of what is ethically correct but institutional and structural constraints prevent the desired course of action." (p.1077). Themes found:<br>1. Resources- lack of time/staff, lack of beds. 2. Rules vs. praxis- difficulty following policy, breaking rules voluntarily & being forced, justifying breaking rules. 3. Conflicts of interest- patients' integrity, professional secrecy & relations, patients vs. colleagues. 4. Lack of supporting structures.
Maiden et al.,[22] USA, quantitative	To examine relationships between MD, compassion fatigue (CF) and perceptions about medication errors. Certified critical care nurses (CCRNs) from members of	MDS, 38-item scale,<br>0-6 scale. Cronbach α = 0.97. The Professional Quality of Life Scale (ProQOL) - to measure CF, 30-item, 0-4 scale. Cronbach α	MD defined as an "individual knowing the correct course of action to take, but because of real or perceived institutional constraint or barrier it is impossible to carry out the correct course of action" (p.341) - provided as a direct quote from Jameton[3]. Moderate statistically significance found between MD and CF

	the American Association of Critical-Care Nurses (ACCN) *(n=205).* 5 CCRNs working clinically also recruited to participate in one focus group.	= 0.81. Medication Administration Error Survey, 77-item from Wakefield et al.[23] to assess nurses' perceptions of why medication errors occur, why they aren't reported & an estimated percentage of actual errors reported. Focus group with 5 CCRNs to discuss medication errors, issues & feelings related to errors & resources to discuss.	*(p<0.001)*- the more MD, the higher their perception of CF. CCRNs reporting intent to resign also reported higher mean CF scores. Higher level of MD associated with perception that lack of communication with physicians cause medication errors. Medication errors similar psychological feelings to MD, cause some CCRNs to consider leaving nursing.
McCarthy & Deady,[24] USA, theoretical	To reconsider the concept of moral distress.	Review of the literature, not systematic.	MD not specifically defined, described as knowing the right thing to do but unable to do it, or doing what is believed to be the wrong thing. 2 concerns regarding the evolution of MD: 1) Currently it is an umbrella term, capturing the experiences of people who are morally constrained, internally & externally. However also concerned that research on MD lacks conceptual clarity & there is an overemphasis on the psychological components & not enough on the ethical. 2) Argue MD perpetuates negative meta-narratives of nurses suffering, powerless and ineffective in moral decision-making. Should not restrict research regarding MD to nurses alone.
Musto & Rodney,[25] Canada, theoretical	No stated aim.	Theoretical- critical realism (CR). Argue that critical realism provides the ideal method in which to further explore and understand MD. Quantitative and qualitative methods are limited as to what they can find and therefore reveal about MD. CR is concerned with ontology- to say something about a thing itself not beliefs, experiences or current knowledge.	There is a lack of conceptual clarity regarding MD definition, study & application. There needs to be revision of the epistemological assumptions underpinning knowledge & use of the concept. Critical issues with the concept: 1. the location of the MD (within the individual or within the structures of healthcare). 2. Jameton's definition links with action & inaction. Argue that we need to understand the interplay between individuals' actions & the structural context, which is rife with power dynamics; researchers commit 'epistemic fallacy'- conflate the concept with the measures to study it. Nurses do not always experience MD when there is a constraint preventing their moral judgement & therefore there must be more behind the concept. They suggest this could be contextual and suggest ontological study of MD.
Nathaniel,[26] USA, qualitative	To further elucidate the experiences & consequences of nurses' moral distress & to formulate a logical, systematic theory of moral distress & its consequences. This is presented as a newly developed grounded theory of moral reckoning in nursing. RNs *(n=21),* not stated where recruited but that the study was advertised in	Grounded theory. Informal, unstructured interviews, not audio-recorded- field notes taken during & immediately after. Line by line coding. Concepts gathered. Constant comparison used to create theory from data. Feedback gained from participants once the theory was developed. Existing literature on MD used to inform theory development.	MD defined as "Moral distress is pain affecting the mind, the body, or relationships that results from a patient care situation in which the nurse is aware of a moral problem, acknowledges moral responsibility, and makes a moral judgment about the correct action, yet, as a result of real or perceived constraints, participates, either by act or omission, in a manner he or she perceives to be wrong." (p.421) MD did not emerge as a major theme instead 'moral reckoning' did- argues this moves beyond MD, explaining the processes of ease, resolution, & reflection- process of moral-decision making. Story-telling emerged as integral.

	newsletters, a conference and shared with nurse leaders to advertise.		
Peter & Liaschenko,[27] Canada, theoretical	To deepen the understanding of MD and moral ambiguity through a spatio-temporal analysis of proximity.	Theoretical argument with empirical literature to support.	MD follows from the fact RNs are situated in a certain ‘social space’ in healthcare, situated in the nurse-patient relationship, which requires physical nearness. RNs have a sense of moral agency and identity for example in unsupportive environments where there is a lack of staffing and lack of recognition, this is threatened & MD results. Proximity to patients means RNs feel their moral responsibilities very acutely & consequently MD. There is moral ambiguity because it is not clear where RNs moral responsibilities begin & end.
Peter & Liaschenko,[28] Canada, theoretical	To explore the concept of moral distress in light of feminist theory.	Theoretical argument with empirical research to support.	Feminist theory offers insight into 3 core elements of moral agency: identities, relationships and responsibilities. MD is a reaction to the constraints on these 3 core elements. 1. MD threatens nurses’ identity as virtuous caregivers. 2. MD represents failure to adhere to normative expectations, personally and institutionally. 3. Nurses responsibilities are enshrined in codes of professional ethics but unlike others, often the realizations of these are dependent on others. Close proximity to patients results in shared suffering and heightened moral distress. To reduce MD they encourage sharing one’s values & responsibilities, repairing damaged identity with stories of resistance & a movement towards relief of suffering.
Peter et al.,[29] Canada, qualitative	To explore nurses’ moral knowledge in cases of perceived overly aggressive care. Graduate RNs *(n=15)* from any area of clinical practice studying at one university invited. Inclusion criteria= those who experienced MD when caring for a patient receiving perceived overly aggressive care.	Critical narrative approach- participants asked to describe the situation in question. Did not define ‘aggressive care’ to allow for open responses. Narratives were audio recorded and transcribed. Narrative thematic analysis. Analysis and themes agreed upon within the research team- the dominant theme was used in creation of the typology. Audit trail utilized and reflexivity employed.	MD defined according to Jameton[3]- authors question what nurses ‘know’ to be the ‘right thing to do.’ 4 narratives identified: 1. ‘Wait and see: medical uncertainty.’ & importance of time to see if interventions work. 2. ‘Deflected responsibilities to respond to dying, death or futility.’- focusing on cures & avoiding difficult conversations, delegating communication. 3. ‘Divergent understanding, responsibilities, & temporalities.’- different faiths, views of familial responsibility & belief in alternative medicine. 4. ‘Privileged medical understandings & responsibilities’-medical dominance & focus on cure prolongs aggressive care.
Redman & Fry,[30] USA, theoretical	To discuss what can be learned from nurses’ ethical conflicts.	Systematic analysis of 5 methodologically similar studies for: 1. Character of the ethical conflict. 2. Similarities & differences in how conflicts were experienced & resolved. 3. Themes within the speciality areas.	The ethical conflicts were experienced as moral distress, moral uncertainty and moral dilemmas- these are defined according to Jameton. A significant number of ethical conflicts were experienced as MD. Resolution was variable, depending on the speciality area of practice. Moral uncertainty was the least experienced.
Reed & Rishel,[31] USA, theoretical	To suggest that epistemic injustice may be an important contributing factor to nurse moral distress & that hospital policy	Conceptual argument, hypothetical case study and empirical literature to support.	Focusing on frontline clinical RNs, the authors ague patients are being nursed longer in ICUs rather than receiving palliative care which places nurses in ethically challenging situations. RNs carry considerable moral burden as the most trusted profession. They are in close proximity to the patient yet may not

	may be one approach to address this problem.		be informed about all of the treatment decisions whilst expected to advocate, especially in end-of-life scenarios- MD is inevitable. This places the nurses in a position of epistemic injustice. Epistemic injustice is of 2 kinds: 'testimonial injustice' & 'hermeneutical injustice'.
Rushton et al.,[32] USA, quantitative	To assess nurse characteristics for burnout, MD & resilience across 6 'high-stress' units. RNs *(n=114)* from 4 hospitals in one health system, pooled from 6 'high-stress' units: 2 paediatric/neonatology, 2 oncology and 2 adult critical care units.	Cross-sectional survey, 6 survey tools (Masclach Burnout Inventory, Moral Distress Scale (Corley's), Perceived Stress Scale, Resilience Scale, Meaning Scale, State Hope Scale) and socio-demographic data. All completed online, taking up to 30 minutes per participant.	MD defined as occurring when "the person is aware of a moral problem, acknowledges moral responsibility, and makes a moral judgement about the correct action; yet as a result of real or perceived constraints participates in perceived moral wrongdoing"(Nathaniel, 2002). Similarity across clinical groups on all 6 survey tools, with the exception of higher levels of self-reported MD in adult critical care staff (mean SD= 69.1) than in neonate/paeds (49.4) & med/surg/oncology (41.8) *p=0.002*.
Thomas & McCullough,[33] USA, theoretical	To classify the philosophical concepts within moral distress into distinct categories- to provide philosophical clarity and guide further empirical & philosophical investigations.	Conceptual analysis, beginning with Jameton's definition, utilizing literature that extends Jameton's definition & that "point toward a philosophical taxonomy of ethically significant moral distress" (p.105).	Jameton's definition consists of 2 key components: 1. Moral knowledge about what one ought to do in specific circumstances. 2. Organisational constraints on implementing that knowledge. They elaborate on 2- arguing that 'institutional constraints' take the form of challenges to, threats to, & violations of professional & individual integrity. These 6 stages are said to provide further evidence of the crescendo effect & moral residue.
Webster & Baylis,[34] USA, theoretical	To discuss the healthcare ethics consultant's experience of moral distress that can lead to compromised moral integrity and moral residue.	Theoretical.	MD defined according to Jameton[3]. Argue his definition is too 'narrow'- distinguishes moral uncertainty from MD, stating that MD occurs "when there is incoherence between one's beliefs and one's action, and possibly also outcomes."(p.218) MD can also occur when one fails to pursue what one believes to be the right course of action, for example due to an error of judgment, personal failing. Introduce the concept of 'moral residue'- when in the face of MD we have "seriously compromised ourselves or allowed ourselves to be compromised- due to error (realisation of error) or a cause of error (incremental loss of commitment to previously held values for reasons of self-interest, i.e. self-protection, self-promotion)."
Whitehead et al.,[35] USA, quantitative	To assess and compare levels of MD amongst HCPs, relationships between MD, perception of ethical climate, intent to leave , education in end of life care (EOL) & pain management. Total HCPs (*n=592)*; RNs(*n=395)*; Physicians (*n=111)*; others HCPs (*n=86)* of which (*n=45)* provide direct patient care (physical therapists, respiratory therapists,	MDS-R from Hamric et al.[2], items reworded for different professions, 0-4 scale, measuring frequency & intensity. Shortened Olson's HECS-S from Hamric & Blackhall [36]. Surveys distributed via a web-based system to all HCP's. Cronbach α 0.90.	Higher MD scores for RNs (M 82.9) & other HCP's involved in direct care (84.1) compared to Drs (65.8) & HCPs in indirect care (47.6). Higher MD in ICU setting (89.0) compared to non-ICU (70.5) *(p=0.008)*. Variability of MD scores within professions. Effects of MD on direct care & indirect caregivers, possible effects of hierarchy for MD and relationship of MD & ethical climate. HEC-S scores negatively correlated with MDS-R mean scores *(p<0.0001)* - higher perceptions of ethical climate were associated with lower MD scores. Physicians had more positive perception of HEC. Participants had

	occupational therapist, speech therapist) and (*n=41)* provide indirect patient care (chaplains, dieticians, pharmacists, social workers) from 1 healthcare system. RR 22%.		considered leaving/ left a previous position had higher MD scores. 20% RNs, 18% Drs, 12% HCPs considering leaving.
Wiegand & Funk,[37] USA, mixed-methods	To identify clinical situations that cause MD, the consequences of those experiences & whether nurses change their practice based on the experience of MD. Critical care nurses *(n=47, although authors state 49 in the abstract)* from one university hospital. Convenience sample of RNs from 6 adult critical care units. First question asked if respondents experienced MD, if yes they continued, 10 had not experienced MD, 37 had & so completed the entire survey.	Open-ended surveys. Exact wording of the survey questions are not provided. The authors asked for a description of a situation that contributed to an experience of MD, how they addressed the situation, effects on the patient, family and healthcare providers & what they might do if faced with a similar situation in the future. Qualitative Data Analysis Software (ATLAS.ti) used to manage data & code themes. Data analysed inductively. 10 random surveys analysed by co-author to check consistency. RR= 23%.	MD defined as "a type of moral conflict that occurs when one knows the right thing to do, but can't pursue the right action."- defined at the top of the survey. 79% experienced MD, 21% had not. Situations causing MD: 73% end of life: 59% medical futility; 11% organ donation; 8% under or overuse of analgesics. One direct example of a participant's experience of MD provided. Negative consequences for patients and family members were described. Personal & professional consequences of MD for RNs listed.
Wilkinson,[8] USA, qualitative	To explore MD as experienced by RN in acute care, to generate theory about the relationship between moral aspects of nursing & the quality of patient care. List of RN's supplied by the State Board of Nursing- a random sample of 3790 RN's were selected & letters of invitation sent out. 26 responses received, but only 24 interviewed- no explanation provided. It emerged that 11 of the RNs were no longer staff nurses, but they decided to interview for comparative data.	Face-to-face interviews *(n=24),* 13 staff nurses from acute care setting, 11 non-staff nurses with experience in acute care. Audio recorded & transcribed. Open-ended questions with prompts. Examples from participants presented verbatim. Few indications of reflexivity but did reflect on their impression of the participants. As 'indicators' emerged they developed 3 models- a 'moral distress equation', 'moral outrage equation' & 'moral distress model' to show the relationship between the indicators- potentially simplistic, linear & reductionist.	MD defined as "the psychological disequilibrium & negative feeling state experienced when a person makes a moral decision but does not follow through by performing the moral behavior indicated by that decision" (p.16). 7 indicators identified that either contribute to, or are influenced by MD:<br>1. Kinds of cases- often related to prolonging life. 2 .Frequency of occurrence. 3 .Contextual constraints. 4. Feelings. 5. Effect of MD on nurses' wholeness. 6. Effect of MD on patient care. 7. Coping behaviours.

References for Supplementary Material

1. Allen R, Judkins-Cohn T, deVelasco R, et al. Moral Distress Among Healthcare Professionals at a Health System. *JONA'S Healthcare Law, Ethics and Regulation*. 2013; 15: 111-8.
2. Hamric A, Borchers C and Epstein E. Development and Testing of an Instrument to Measure Moral Distress in Healthcare Professionals. *AJOB Primary Research*. 2012; 3: 1-9.
3. Jameton A. *Nursing Practice: The Ethical Issues*. Englewood Cliffs, NJ: Prentice Hall, 1984.
4. Astbury JL, Gallagher CT and O'Neill RC. The issue of moral distress in community pharmacy practice: background and research agenda. *International Journal of Pharmacy Practice*. 2015; 23: 361-6.
5. Barlem ELD and Ramos FRS. Constructing a theoretical model of moral distress. *Nursing Ethics*. 2015; 22: 608-15.
6. Campbell SM, Ulrich CM and Grady C. A Broader Understading of Moral Distress. *The American Journal of Bioethics*. 2016; 16: 2-9.
7. Corley MC. Moral Distress of Critical Care Nurses. *American Journal of Critical Care*. 1995; 4: 280-5.
8. Wilkinson JM. Moral Distress in Nursing Practice: Experience and Effect. *Nursing Forum*. 1987/1988; 23: 16- 29.
9. Corley MC, Minick P, Elswick R and Jacobs M. Nurse Moral Distress and Ethical Work Environment. *Nursing Ethics*. 2005; 12: 381- 90.
10. McDaniel C. Development and psychometric properties of the Ethics Environment Questionnaire. *Medical Care*. 1997; 35: 901-14.
11. Dzeng E, Colaianni A, Roland M, et al. Moral Distress Amongst American Physician Trainees Regarding Futile Treatments at the End of Life: A Qualitative Study. *Journal of General Internal Medicine*. 2016; 31: 93-9.
12. Epstein E and Hamric A. Moral Distress, Moral Residue, and the Crescendo Effect. *Journal of Clinical Ethics*. 2009; 20: 330-42.
13. Fourie C. Moral Distress and Moral Conflict in Clinical Ethics. *Bioethics*. 2015; 29: 91-7.
14. Hamric AB. A Case Study of Moral Distress. *Journal of Hospice and Palliative Nursing*. 2014; 16: 457- 63.
15. Hanna DR. Moral distress: the state of the science. *Research and Theory for Nursing Practice: An International Journal*. 2004; 18.
16. Hanna DR. The Lived Experience of Moral Distress: Nurses Who Assisted With Elective Abortions. *Research and Theory for Nursing Practice: An International Journal*. 2005; 19: 95-124.
17. Hardingham L. Integrity and moral residue: nurses as partiicpants in a moral community. *Nursing Philosophy*. 2004; 5: 127-34.
18. Jameton A. Dilemmas of Moral Distress: Moral responsibility and Nursing Practice. *AWHONNS Clincial Issues in Perinatal and Womens Health Nursing*. 1993; 4.
19. Jameton A. A Reflection on Moral Distress in Nursing Together With a Current Application of the Concept. *Bioethical Inquiry*. 2013; 10: 297- 308.
20. Johnstone MJ and Hutchinson A. 'Moral distress'- time to abandon a flawed nursing construct? *Nursing Ethics*. 2013; 001: 1-10.

21. Kälvemark S, Höglund AT, Hansson MG, Westerholm P and Arnetz B. Living with conflicts-ethical dilemmas and moral distress in the healt care system. *Social Science & Medicine*. 2004; 58: 1075-84.
22. Maiden J, Georges JM and Connelly CD. Moral Distress, Compassion Fatigue, and Perceptions About Medication Errors in Certified Critical Care Nurses. *Dimensions of Critical Care Nursing*. 2011; 30: 339- 45.
23. Wakefield BJ, Uden-Holman T and Wakefield DS. Development and Validation of the Medication Administration Error Reporting Survey. In: Henriksen K, Battles JB, Marks E and Lewin DI, (eds.). *Advances in Patient Safety: From Research to Implementation Vol 4: Programs, Tools, and Products*. Rockville, MD.: Agency for Healthcare Research and Quality AHRQ Publication 05-0021-4., 2005.
24. McCarthy J and Deady R. Moral Distress Reconsidered. *Nursing Ethics*. 2008; 15: 254-62.
25. Musto L and Rodney P. Moving from conceptual ambiguity to knowledgeable action: using a critical realist approach to studying moral distress. *Nursing Philosophy*. 2015; 17: 1-13.
26. Nathaniel A. Moral Reckoning in Nursing. *Western Journal of Nursing Research*. 2006; 28: 419-38.
27. Peter E and Liaschenko J. Perils of proximity: a spatiotemporal analysis of moral distress and moral ambiguity. *Nursing Inquiry*. 2004; 11: 218-25.
28. Peter E and Liaschenko J. Moral Distress Reexamined: A Feminist Interpretation of Nurses' Identities, Relationships, and Responsibilities. *Bioethical Inquiry*. 2013; 10: 337-445.
29. Peter E, Mohammed S and Simmonds A. Narratives of aggressive care: Knowledge, time, and responsibility. *Nursing Ethics*. 2014; 21: 461-72.
30. Redman BK and Fry ST. Nurses' Ethical Conlficts: What is really known about them? *Nursing Ethics*. 2000; 7: 360-6.
31. Reed PG and Rishel CJ. Epistemic Injustice and Nurse Moral Distress: Perspectove for Policy Development. *Nursing Science Quarterly*. 2015; 28: 241- 4.
32. Rushton CH, Batcheller J, Schroeder K and Donohue P. Burnout and Resilience Among Nurses Practicing in High-INtensity Settings. *American Journal of Critical Care (AJCC)*. 2015; 24.
33. Thomas T and McCullough L. A Philosophical Taxonomy of Ethically Significant Moral Distress. *Journal of Medicine and Philosophy*. 2015; 40: 102-20.
34. Webster GC and Baylis FE. Moral Residue. In: Rubin SB and Zoloth L, (eds.). *Margin of Error: The Ethics of Mistakes in the PRactice of Medicine*. Hagerstown, Maryland: University Publishing Group, 2000.
35. Whitehead PB, Herbertson RK, Hamric AB, Epstein EG and Fisher JM. Moral Distress Among Healthcare Professionals: Report of an Institution-Wide Survey. *Journal of Nursing Scholarship*. 2014; 47: 117-25.
36. Hamric A and Blackhall L. Nurse-physician perspectives on the care of dying patients in intensive are units: Collaboration, moral distress, and ethical climate. *Society of Critical Care Medicine*. 2007; 35: 422-9.
37. Wiegand DL and Funk M. Consequences of clinical situations that cause critical care nurses to experience moral distress. *Nursing Ethics*. 2012; 19: 479- 87.
